# A Monte Carlo Permutation Test for Random Mating Using Genome Sequences

**DOI:** 10.1371/journal.pone.0071496

**Published:** 2013-08-05

**Authors:** Ran Li, Minxian Wang, Li Jin, Yungang He

**Affiliations:** 1 Chinese Academy of Sciences and Max Planck Society (CAS-MPG) Partner Institute for Computational Biology, Shanghai Institutes for Biological Sciences, Chinese Academy of Sciences, Shanghai, China; 2 Key Laboratory of Computational Biology, Chinese Academy of Sciences and Max Planck Society (CAS-MPG) Partner Institute for Computational Biology, Chinese Academy of Sciences, Shanghai, China; 3 State Key Laboratory of Genetic Engineering and Ministry of Education Key Laboratory of Contemporary Anthropology, School of Life Sciences and Institutes of Biomedical Sciences, Fudan University, Shanghai, China; Cincinnati Children's Hospital Medical Center, United States of America

## Abstract

Testing for random mating of a population is important in population genetics, because deviations from randomness of mating may indicate inbreeding, population stratification, natural selection, or sampling bias. However, current methods use only observed numbers of genotypes and alleles, and do not take advantage of the fact that the advent of sequencing technology provides an opportunity to investigate this topic in unprecedented detail. To address this opportunity, a novel statistical test for random mating is required in population genomics studies for which large sequencing datasets are generally available. Here, we propose a Monte-Carlo-based-permutation test (MCP) as an approach to detect random mating. Computer simulations used to evaluate the performance of the permutation test indicate that its type I error is well controlled and that its statistical power is greater than that of the commonly used chi-square test (CHI). Our simulation study shows the power of our test is greater for datasets characterized by lower levels of migration between subpopulations. In addition, test power increases with increasing recombination rate, sample size, and divergence time of subpopulations. For populations exhibiting limited migration and having average levels of population divergence, the statistical power approaches 1 for sequences longer than 1Mbp and for samples of 400 individuals or more. Taken together, our results suggest that our permutation test is a valuable tool to detect random mating of populations, especially in population genomics studies.

## Introduction

In a random mating population all individuals have an equal chance of being a mating partner. In population genetics, deviations from random mating may indicate inbreeding, population stratification, natural selection or sampling bias. Extensive association studies have been conducted on population samples to search for genes underlying complex traits through linkage disequilibrium of these genes with markers [1–8]. However, when samples originate from a non-random mating population, spurious associations may arise between marker loci and complex traits. In evolutionary studies, it is important to determine whether a given locus is under random mating since deviations may be due to natural or artificial selection [9]. In population genetics, samples are usually tested to determine if they are derived from the same random mating population [9], since the samples might exhibit signs of genetic stratification even if they are from one locality.

Many methods have been proposed to test for random mating. They can be divided into two categories: asymptotic and exact tests. Several asymptotic tests (also know as “goodness-of-fit” or*χ* ^2^ tests) have been developed based on asymptotic theory. They perform well when considering independent loci having a small number of alleles [10–12]. However, for loci having a large number of alleles, the contingency table used to implement asymptotic tests usually contains too many empty cells and the number of individuals in the sample is insufficient for large sample theory to be applied [13–24]. Although the “single allele test” addresses the problem of sparse tables by analyzing each allele separately, the statistical power of this approach is limited because multiple comparisons are made [9]. With the advent of dense genome-wide sequencing, loci having large numbers of alleles, and the genome as a whole, are now available for populations genomics investigation [25]. These studies generate sparse-matrix data for which asymptotic methods are not reliable. In such cases, exact methods are necessary.

Exact tests use the exact probability of potential outcomes rather than using an asymptotic probability distribution. The p-value of the exact test is given by the sum of the exact probabilities of the allele combinations that deviate from the null hypothesis of random mating by at least as much as the observed sample. The idea of the exact test was first proposed by Fisher (1935) and subsequently advocated by Levene (1949) and Haldane (1954) in genetics [26,27]. However, the application of the exact test was hindered by its computational complexity until Louis and Dempster (1987) proposed a complete enumeration algorithm to compute the p-value for this test [15]. Unfortunately, this method is computationally impractical when the number of alleles is large. This prompted the development of Monte Carlo (MC) and Markov chain Monte Carlo (MCMC) methods, which are easy to perform and have become widely used in population genetics [16,17].

In addition to the above statistical tests, some other methods, such as STRUCTURE analysis and PCA analysis, can be used to infer possible genetic substructure of populations, thus providing evidence of random mating [4,28–30]. However, these methods cannot act as a substitute for statistical tests of random mating.

Neither asymptotic nor exact tests possess enough statistical power to take advantage of the large amount of polymorphism data made available by genome-wide sequencing. Therefore, there is considerable interest in novel statistical tests for detecting random mating using large-scale sequencing data. In this study, we address the shortcomings of the existing methods by developing a Monte-Carlo-based-permutation (MCP) test to detect random mating. Using computer simulations, we demonstrate that the MCP test performs well on large-scale sequencing data and that its statistical power is greater than that of the classical chi-square test (CHI test). Here, we discuss the influence of genetic and demographic parameters, such as sequence length, sample size, mutation rate, recombination rate, divergence time, and migration rate, on the performance of the MCP test.

## Materials and Methods

### Model of random mating for sequence data

A random mating population is one in which all individuals have the same probability of being mating partners. In other words, potential mates have an equal chance of being chosen, without being influenced by environmental, hereditary, or social factors. In this context, the process of random mating can be treated as a random sampling process. In our MCP method, we simulated random mating as the pairing of sequences from randomly selected individuals. Random mating was simulated for a sample of individuals as follows: (i) two gametes from these individuals, which were not necessarily from the same individual, were randomly chosen without replacement to generate a new individual; (ii) further two gametes were then randomly chosen from the remaining pool of gametes to generate another new individual; (iii) this process was repeated until every gamete had been chosen. By treating this process as a simple Monte Carlo permutation procedure, individuals from a random mating population were simulated. After choosing an appropriate statistic, the null distribution of this statistic from a random mating sample can be obtained. By comparing the observed statistic to its null distribution, standard hypothesis testing can be performed to determine if the sample is derived from a random mating population. This approach resembles an exact test which randomly samples alleles [17].

### Average Pairwise difference within individuals as a statistic

Pairwise difference, denoted by ξ in this study, is the number of different nucleotides between aligned sequence pair. The expected pairwise difference for a pair of sequences (*E*(*ξ*)) is proportional to the mutation rate (*μ*) and expected coalescence time (*T*) for that pair of sequences (i.e.*E*(*ξ*)=2*μT*). In a population under random mating conditions, the expected pairwise difference is the same for all randomly selected pairs of sequences. However, for a non-random mating population, the expected pairwise differences for randomly selected pairs of sequences differ according to the population substructure. For simplicity, we assume that sequences are sampled from two homogeneous subpopulations called A and B. *T*_*AB*_ denotes the expected coalescence time of any pair of sequences, of which one sequence comes from subpopulation A, the other from B. Similarly, *T*_*AA*_ and *T*_*BB*_ denote the expected coalescence time of any two sequences both coming from subpopulation A or B, respectively. *ξ*_*AB*_ is the pairwise difference of two sequences coming from two different subpopulations, and *ξ*_*AA*_, *ξ*_*BB*_ are the pairwise differences when both come from subpopulation A or B, respectively. We can infer that *E(ξ*_*AB*_ *)* > *E(ξ*_*AA*_ *)* and *E(ξ*_*AB*_ *)* > *E(ξ*_*BB*_ *)* in the case of a non-random mating population, since *T*_*AB*_ >*T*_*AA*_ and *T*_*AB*_ >*T*_*BB*_.

We suppose that a sample of size n individuals is drawn from a population of interest. Sequences of the individuals are denoted by C_1_, C_2_…, to C_2n_ where C_1_ and C_2_ are from individual 1, C_3_ and C_4_ are from individual 2, and so on. Under random mating, a sample of size n has an observed average pairwise difference within individuals as defined by: 

ξ_observe_=(ξ_C1C2_ + ξ_C3C4_ +……+ξ_C(2n-1)C(2n)_ )*/n*,

where *ξ*_*C*1*C*2_ is the pairwise difference between sequences C_1_ and C_2_, and so on. When these sequences are randomly permuted and divided into n pairs, we obtain a simulated sample where the average pairwise difference within individuals is defined as 

ξ_permute_=(ξ_Cs1Cs2_ + ξ_Cs3Cs4_ +……+ξ_Cs(2n-1)Cs(2n)_ )*/n*,

where, *s*_1_, *s*_2_…, until *s*_2*n*_ is an order of one permutation of sequence 1 to 2n. When a sample of sequences is collected from a random mating population, *E(ξ*_*observe*_ *)* = *E(ξ*_*permute*_ *)*, since all sequence pairs have the same expected coalescence time. However, if sequences are chosen from a non-random mating population, containing two subpopulations A and B, there are three possible pairwise differences for the simulated samples: ξ_AA_, ξ_BB_, and ξ_AB_. Thus *ξ*_*permute*_ is a combination of these three different pairwise differences, whereas in the real sample, *ξ*_*observe*_ is only a combination of ξ_AA_ and ξ_BB_. Because *E(ξ*_*AB*_ *)* > *E(ξ*_*AA*_ *)* and *E(ξ*_*AB*_ *)* > *E(ξ*_*BB*_ *)*, it follows that *E(ξ*_*permute*_ *)* > *E(ξ*_*observe*_ *)*. Therefore, the test of whether the sample is from a random mating population can be formulated as

H_0_: ξ_observe_ = ξ_permute_

H_1_: ξ_observe_ < ξ_permute_

According to the null hypothesis (H_*0*_), the sample is from a population under random mating whereas according to the alternative hypothesis (H_*1*_), the population is not randomly mating.

### Hypothesis testing

**Figure 1:**
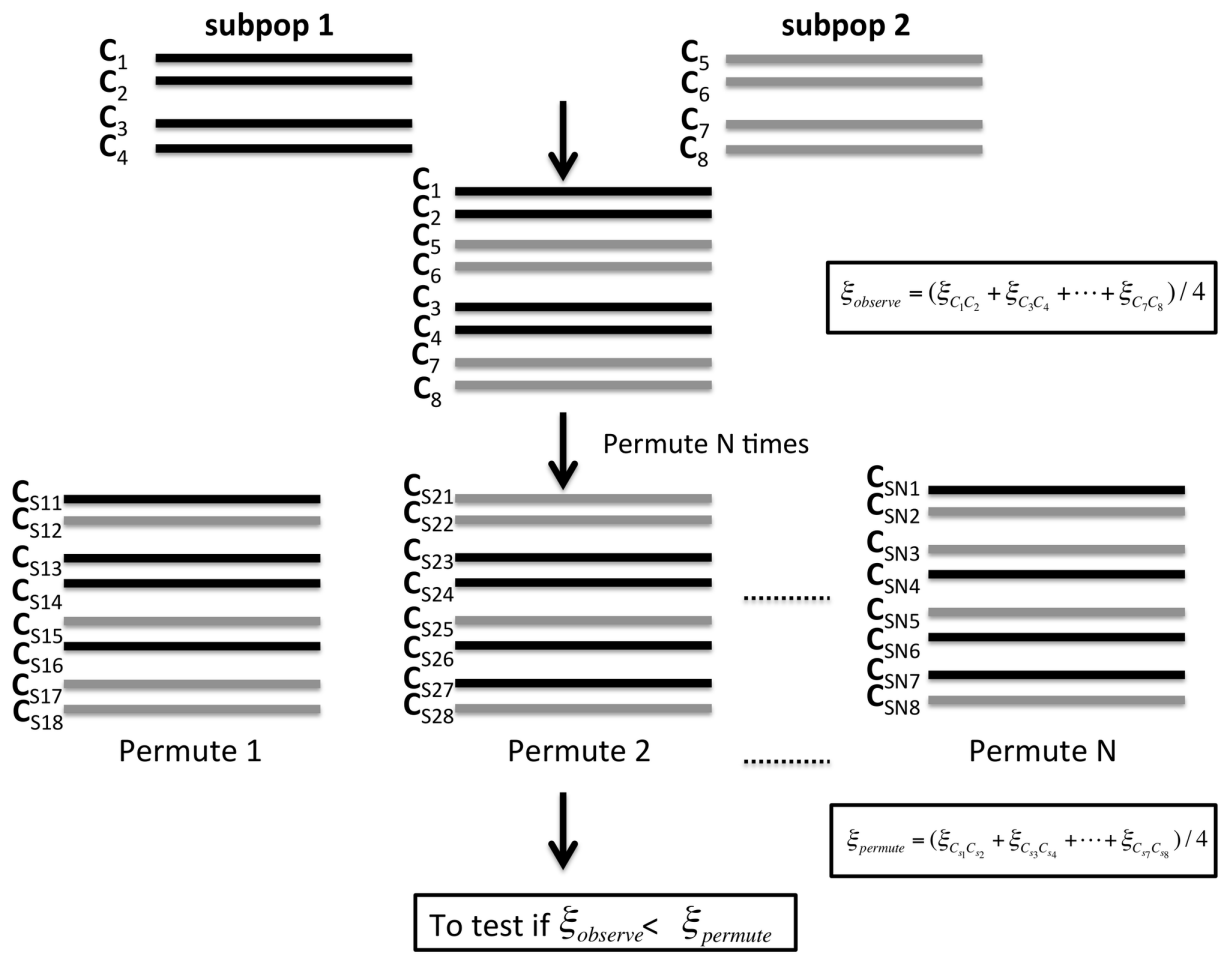
A diagram of the MCP method. In this case, a sample of n = 4 individuals was drawn from a population of interest. Gamete sequences (2n) of the individuals are denoted by C_1_-C_8_ as follows: C_1_ and C_2_ are from individual 1, C_3_ and C_4_ are from individual 2 and so on. After permuting these sequences N times (where N is any positive integer), N new datasets were obtained by dividing each permuted sequence into n consecutive pairs. For each permutation, the *ξ*_*permute*_ statistic could be calculated. This allowed us to derived the null distribution of the statistic. After locating *ξ*_*observe*_ on the null distribution, a p value of the test could be obtained.

Under the null hypothesis of random mating, the distribution of average pairwise sequence differences within individuals is equivalent to that of a simulated sample obtained by the permutation procedures described above. In statistical hypothesis testing, many permutations are conducted to obtain the null distribution of average pairwise differences within individuals. In other words, the null distribution of *ξ*_*observe*_ can be obtained by calculating *ξ*_*permute*_ for each of the simulated samples generated in the permutation procedure. For a given permutation test, the significance level (p-value) of the null hypothesis is the probability that *ξ*_*permute*_ is equal to, or less than, *ξ*_*observe*_. The hypothesis testing procedure is graphically outlined in Figure 1.

To ensure that the obtained p-value is within δ units of the true significance level, at a (1-γ) % confidence level, the required number of permutations is given by$N\geq {\left(\frac{Z_{\gamma /2}}{2\delta }\right)}^{2}$, where *Z* is the z-score of a standard normal distribution [31]. In this case, for *δ*=0.01 and a confidence level of 99%, $N\geq {\left(\frac{2.576}{2\delta }\right)}^{2}\approx 17000$ permutations are sufficient. Therefore, to obtain reliable p-values in the present study, we performed 20,000 permutations for each statistical hypothesis test.

### Performance evaluation

To evaluate the performance of our statistical test for random mating, simulated genomic sequences in multiple genetic scenarios were generated using the MS software program [32]. Both type I error rate (i.e. false positive rate) and type II error rate (i.e. 1-statistical power) were evaluated. Experimental parameters (e.g. sample size *n* and sequence length *l*), and inherent parameters (e.g. mutation rate and recombination rate), may affect the type I error of the MCP test. Under the alternative hypothesis that mating is non-random, the power of this test may also be affected by two parameters related to demographic history: the divergence time of the subpopulations and the migration rate between them.

Simulated datasets of genomic sequences were generated using the “control of variables” strategy [33]. To evaluate how a specific parameter affects the type I or type ΙI error of the MCP test, the parameter’s values were varied in the simulation while all other parameters were kept constant (we refer to these as “steady states”) (Table 1). For example, we tested how sequence length affects the performance of MCP when all other parameters (e.g. sample size, recombination rate, and mutation rate) were kept in a “steady state”. In each case, 1000 replicates were generated, thus yielding 1000 p-values in statistical tests for which type I and type II errors could be examined. Notations used in this report and the values of parameters in “steady states” are presented in Table 1.

**Table 1:** Parameters and terminology.

Symbols	Explanation	“Steady states”
N	Effective population size	5000
r	Recombination rate per site per generation	10^-8^
*l*	Sequence length	1Mbp
μ	Mutation rate (per generation per site)	10^-8^
*T*	Divergence time of two subpopulations (generations).	400
*n*	Sample size (number of individuals)	400
θ	4Nμ*l*	200
ρ	4Nr*l*	200
*m*	Migration rate per generation	0
*M*	4N*m*	0
β	Significance level	0.05 or 0.01

## Results

### Evaluation of type I error

To evaluate the influence of experimental and inherent parameters on the MCP test, we estimated the type I error rate of the MCP test by simulations in which individual parameters were varied using the “control of variables” strategy (see Materials and Methods for details). In these simulations, sequence length was varied from 5kb to 2Mb and sample size was varied from 50 to 800 individuals. Since different genome regions differ in their recombination (*ρ=4Nrl*) and mutation rates (*θ=4Nμl*), we assessed both to evaluate their influence on the type I error of our method (Table 2).

**Table 2:** Type 1 error of the MCP method with different parameters.

Significance levels	*l* = 5kb	*l* = 50kb	*l* = 500kb	*l* =1Mb	*l* = 2Mb
0.05	0.034	0.054	0.041	0.069	0.048
0.01	0.004	0.016	0.004	0.016	0.015
Significance levels	*n* = 50	*n* = 100	*n*= 200	*n* =400	*n* =800
0.05	0.027	0.048	0.048	0.048	0.054
0.01	0.0047	0.011	0.012	0.011	0.017
Significance levels	ρ = 100	ρ = 200	ρ = 400	ρ = 800	ρ = 1000
0.05	0.048	0.051	0.039	0.043	0.038
0.01	0.004	0.011	0.010	0.007	0.005
Significance levels	θ = 100	θ= 200	θ= 400	θ= 800	θ= 1000
0.05	0.054	0.057	0.043	0.042	0.061
0.01	0.010	0.012	0.008	0.004	0.010

We used control variable strategy to detect type Ι error of MCP test in different sequence length *l*, sample size *n*, recombination rate ρ = 4Nr*l* and mutation rate θ = 4Nμ*l*, corresponding to two significance levels 0.05 and 0.01. When detecting the effects of one specific parameter, the values of the other parameters kept in “steady states” were as follows: sequence length *l*= 1Mbp; effective population size N=5000; recombination rate ρ=4Nrl=4×5000×10^-8^ *l* ; sample size *n*=400 individuals from a random mating population and mutation rate θ=4Nμl=4×5000×10^-8^ *l*.

Our simulations indicate that type Ι error is well controlled in the MCP test (Table 2). At a significance level of 0.05, type I error rate ranged from 0.027 (for *n*=50) to 0.069 (for *l*=1Mb) (Table 2). In simulations of the MCP test, under the null hypothesis of a randomly mating population, the number of the p-values smaller than a threshold *p* followed a binomial distribution **B**(*m*, *p*), where *m* is the number of replicates. Therefore, when *m*=1000 and *p*=0.05, 95% of estimated type I error rates are expected to lie in the range 0.0373 to 0.0654. In our evaluations, all the estimated type I error rates lie in this range, except for the two extreme cases noted above. Furthermore, when *m*=1000 and *p*=0.01, 95% of the estimations are expected to fall between 0.0048 and 0.0183. In this study, most of the corresponding estimations fell into the expected range and none of them exceeded the upper boundary (Table 2). Test for type I error for more scenarios are presented in Tables S1-S4.

### Evaluation of statistical power

Given the generally favorable evaluation of type Ι error rate, we sought to examine the statistical power of the MCP test at significance levels of 0.05 and 0.01. We considered experimental, inherent, and demographic parameters under the alternative hypothesis to determine their effect on statistical power.

We compared our MCP test to the CHI test. Since the CHI test uses numbers of genotypes and alleles, it cannot be directly implemented using real sequences. Therefore, we chose a fixed number of equally distanced SNPs, treating them as independent loci. We also evaluated the influence of locus number (from 1 to 100 in increments of 10) on the performance of the CHI test. When *n* loci (*n*>1) were available in the CHI test, we calculated Pearson’s chi-statistic for each locus and summarized this to obtain a summary statistic following a standard central chi-square distribution with *n* degrees of freedom [34]. In simulations, we found that the type Ι error rate of the CHI test was greater than expected, which may be due to the interdependence of the loci involved (Tables S5-S8). To compensate for the inflated type I error in the CHI test power evaluation, we replaced the standard rejection criteria with empirical thresholds for significance levels 0.01 and 0.05, represented respectively by the 10^th^ and 50^th^ ranked values of the CHI test summary statistic in 1000 simulated tests under the null hypothesis. This allowed us to calculate the statistical power of the CHI test at different loci. The highest statistical power of the CHI test using different numbers of loci was chosen for comparison to our method (Tables S9-S12).

**Figure 2:**
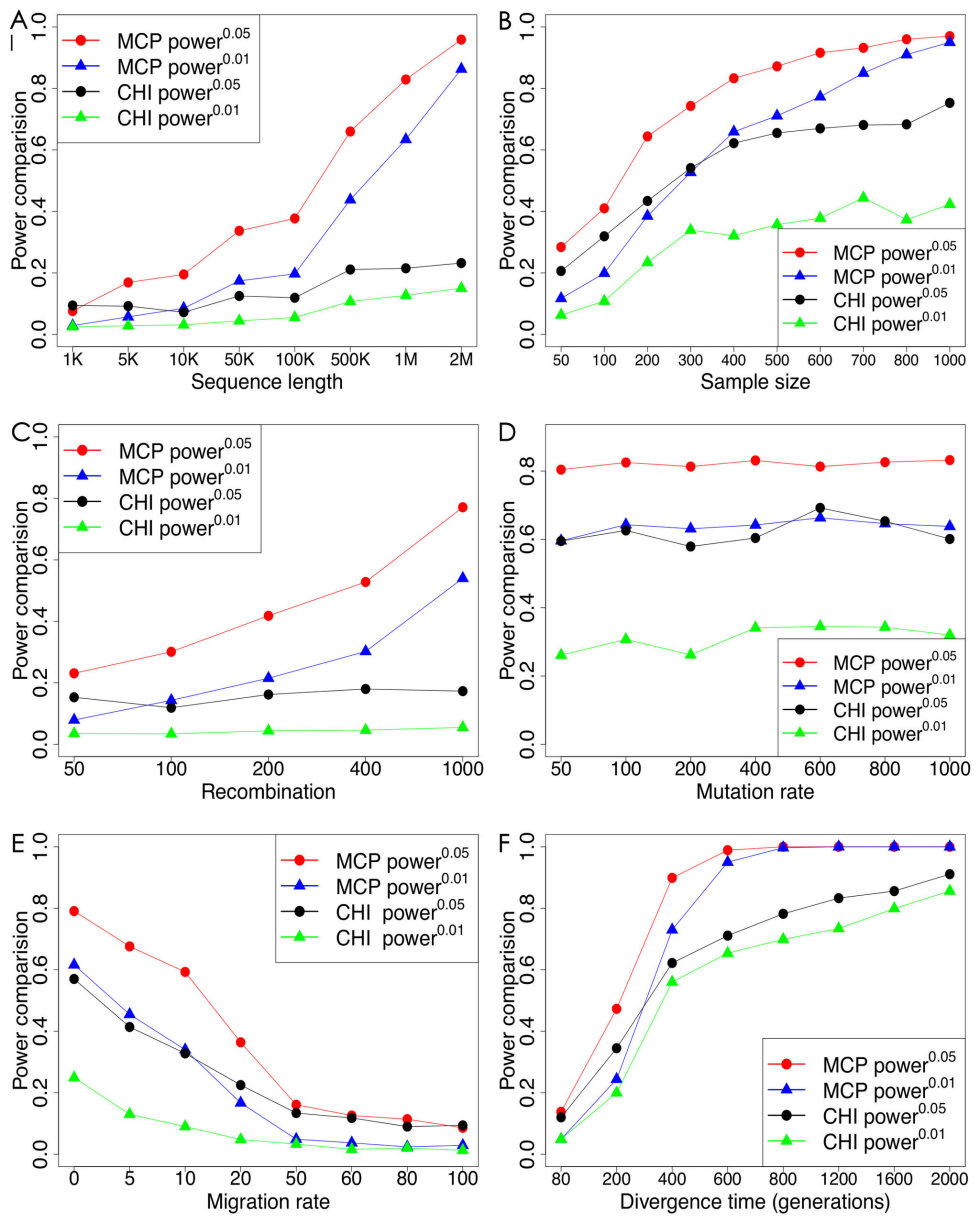
Statistical power of MCP and CHI tests under varying: (A) sequence length, (B) recombination rate, (C) sample size, (D) migration rate, (E) population divergence, and (F) mutation rate.

Our investigation showed that the MCP test has more statistical power than the CHI test in experimental designs with different sequence lengths and sample sizes. For sequence length, the statistical power of the MCP and CHI tests was compared for eight lengths, in the range of 1kb to 2Mb (Figure 2A). The statistical power of both tests increased with an increase in sequence length, however, the power of the MCP test was consistently much higher than that of the CHI test. For example, when *l*=1Mbp, the power of the MCP test reached 0.8 or higher, whereas the power of the CHI test was only around 0.2. Furthermore, for sequence lengths greater than 1.5Mb, the power of the MCP test exceeded 0.9.

For sample sizes ranging from 100 to 1000 individuals, the power of both tests increased with larger sample size, with the power of the MCP test consistently greater than that of the CHI test. For samples of more than 400 individuals, the power of the MCP test exceeded 0.8 for a significance level of 0.05, but the power of the CHI test never exceeded this value, even for sample sizes greater than 1000 individuals (Figure 2B).

The MCP test outperformed the CHI test in genome regions subject to a variety of mutation and recombination rates. We found that statistical power did not vary for mutation rates θ ranging from 50 to 1000 (Figure 2C). However, at a significance level of 0.05, the power of the MCP method consistently exceeded 0.8, whereas the power of the CHI test was approximately 0.6. Furthermore, at a significance level of 0.01, the power of the MCP test was approximately 0.6, whereas that of the CHI test was only 0.3. The power of both tests at different recombination rates ρ ranging from 50 to 1000 was evaluated (Figure 2D). The power of the MCP test increased with increasing recombination rate, whereas that of the CHI test remained relatively constant. With a recombination rate per generation per site of 10^-8^ (ρ=200 when μ=10^-8^), which is the most commonly used recombination rate [35–38], the power of the MCP test exceeded 0.4, but that of the CHI test was less than 0.2, at a significance level of 0.05.

We further compared the statistical power of the MCP and CHI tests in demographic scenarios having different population divergence times and gene migration rates (Figure 2E and Figure 2F). Statistical power increased with divergence time for both methods. Interestingly, for a population divergence of 400 generations, the power of the MCP test was greater than 0.9 at a significance level of 0.05, and approached 1 when population divergence increased to 600 generations whereas the power of the CHI test never exceeded 0.8. The power of both methods was highly dependent on the migration rate between subpopulations. The power was high for small or no migration rates, but declined with increasing migration rate, resulting in no power at the highest migration rates (Figure 2F).

## Discussion

Here we report a Monte Carlo permutation-based (MCP) statistical test for detecting random mating in a population of interest. Computer simulation showed that the type Ι error behaved well in the MCP testing and statistical power of the method compared favorably with the CHI test in most cases. Moreover, this method can be used not only to detect population stratification of genetic samples, but also to test for random mating at specific regions of the genome or multiple tightly linked loci.

Using the average pairwise difference within individuals has the advantage of allowing the MCP test to consider multiple loci without assuming independence between them, since recombination has no effect on this measure in a homogenous population. Linkage disequilibrium (LD), which can even occur between loci situated several kilobases apart, can inflate the type Ι error in statistical tests. Accordingly, we found that the type I error for the CHI test was highly inflated when more markers were used in order to increase statistical power (Tables S5-S8). In inference-based approaches for detecting population stratification, LD is also problematic. For example, STRUCTURE does not fully eliminate the effects of strong LD, which may produce inaccurate results [39–41]. Therefore, it was suggested that loci used as input for STRUCTURE analysis should be separated by at least 1 cM [39–41]. However, this constraint is an obvious drawback in the analysis of genome-wide sequencing data.

In contrast to other methods for detecting random mating [15,27], we found that the performance of our MCP method is not diminished by increasing the number of alleles or haplotypes. In fact, higher statistical power is achieved with longer sequence lengths. Moreover, power increases rapidly especially in cases with larger sample sizes, lower migration rates, higher recombination rates, and larger divergence time between subpopulations (Figure 2). When the inherent genetic and demographic parameters are fixed, higher statistical power of the MCP test can be achieved by increasing the sample size or sequence length. In the worst case, when only a single polymorphic locus is available for random mating tests, the statistical power of the MCP test is still comparable to that of the classical CHI test. Hence, the MCP test is preferable to the CHI test for a broad range of genetic studies. Obviously, false positive result of CHI test is well controlled in scenario with only unlinked loci. Therefore, CHI test is still a good choice in such scenario.

The MCP test is presented in this report as a single-tailed test, rather than a two-tailed test, since inbreeding is common in population genetic history whereas outbreeding is relatively rare [42,43]. Notably, the MCP test can be conveniently modified to form a two-tailed test when necessary. However, a two-tailed version of the MCP test is likely to be impractical for quality control of sequencing projects. This is because when sequencing errors are randomly introduced into sequence products, the errors have little impact on the average pairwise difference. Therefore, a two-tailed MCP test may lack power in detecting sequencing errors. Furthermore, a two-tailed MCP test may not be a good choice for detecting balancing selection because detecting extra heterozygosity has been suggested to be lack of power in simulation study [44,45].

The time complexity of the entire MCP test calculation process is O(mn^2^ +Nn), where m is the number of SNPs, n is the number of individuals and N is the number of permutations. On a cluster machine with 4G RAM, with 4 CPU cores (Dual-Core AMD Opteron(tm) Processor2214, 2194 MHz), with each test executed using a single CPU, it would require about 10 minutes with 2000 individuals and a window size of 2Mbp, using a R script. Thus, the MCP test is suitable for large data sets.

## Supporting Information

Table S1 **We detected type 1 error of the MCP test in different sequence length l corresponding to two different significance levels 0.05 and 0.01.**
Other parameters in “steady states” were as follows: sample size n=400 individuals; effective population size N=5000; recombination rate ρ = 4Nrl=4×5000×10-8l; mutation rate θ = 4Nμl=4×5000×10-8l.
(DOCX)

Table S2 **We detected type 1 error of the MCP test in different sample size n corresponding to two different significance levels 0.05 and 0.01.**
Other parameters in “steady states” were as follows: sequence length l = 1Mb; effective population size N=5000; recombination rate ρ=4Nrl=4×5000×10-8×106=200; mutation rate θ=4Nμl=4×5000×10-8×106=200.
(DOCX)

Table S3 **We detected type 1 error of the MCP test in different recombination ρ corresponding to two different significance levels 0.05 and 0.01.**
Other parameters in “steady states” were as follows: sequence length l = 1Mb; effective population size N=5000; mutation rate θ=4Nμl=4×5000×10-8=200; sample size n=400 individuals.
(DOCX)

Table S4 **We detected type 1 error of the MCP test in different mutation rate θ corresponding to two significance different levels 0.05 and 0.01.**
Other parameters in “steady states” were as follows: sequence length l = 1Mb; effective population size N=5000; recombination rate ρ=4Nrl=4×5000×10-8×106=200, sample size n=400 individuals.
(DOCX)

Table S5 **We compared the type Ι error rate of the MCP test with the CHI test in different recombination rate ρ corresponding to two different significance levels 0.05 and 0.01.**
Other parameters in “steady states” were as follows: sequence length l = 1Mb; effective population size N=5000; mutation rate θ=4Nμl=4×5000×10-8×106=200; sample size n=400 individuals.
(DOCX)

Table S6 **We detected the type 1 error of the CHI test in different sequence length l with certain numbers of loci.**
The longer the sequences, the more loci we could use. Empty cells meant we did not do the experiments because of limited SNPs. Other parameters in “steady states” were as follows: sample size n=400 individual from a random mating population; effective population size N=5000; mutation rate θ=4Nμl=4×5000×10-8×106=200; recombination rate ρ=4Nrl=4×5000×10-8×106=200.
(DOCX)

Table S7 **We detected the type 1 error of the CHI test in different sample size n with certain numbers of loci.**
Other parameters in “steady states” were as follows: sequence length l = 1Mbp; effective population size N=5000; mutation rate θ=4Nμl=4×5000×10-8×106=200; recombination rate ρ=4Nrl=4×5000×10-8×106=200.
(DOCX)

Table S8 **We detected the type 1 error of the CHI test in different mutation rate with certain numbers of loci.**
Other parameters in “steady states” were as follows: sequence length l = 1Mbp; sample size n=400 individuals from a random mating population; effective population size N=5000; recombination rate ρ=4Nrl=4×5000×10-8×106=200.
(DOCX)

Table S9 **We detected the power of CHI test in different migration rate M=4Nm with certain numbers of loci.**
(m is the fraction of each subpopulation made up of new migrants each generation.) Other parameters in “steady states” were as follows: sample size n=400 individuals, in which half of them came from subpopulation 1 and the other half came from subpopulation 2; sequence length l = 1Mbp; effective population size N=5000; mutation rate θ=4Nμl=4×5000×10-8×106=200; divergence time T = 10000 years, recombination rate ρ=4Nrl=4×5000×10-8×106=200.
(DOCX)

Table S10 **We detected the power of the CHI test in different sequence length l with certain numbers of loci.**
Empty cells meant we did not do the experiments because of limited SNPs. Other parameters in “steady states” were as follows: sample size n=400 individuals, in which half of them came from subpopulation 1 and the other half came from subpopulation 2; effective population size N=5000; mutation rate θ = 4Nμl=4×5000×10-8l; divergence time T = 10000 years, recombination rate ρ = 4Nrl=4×5000×10-8l and no migration.
(DOCX)

Table S11 **We detected the power of the CHI test in different sample size n with certain numbers of loci.**
Other parameters in “steady states” were as follows: sample size n=400 individuals, in which half of them came from subpopulation 1 and the other half came from subpopulation 2; the divergence time of the two subpopulations T=10000 years; effective population size N=5000; mutation rate θ=4Nμl=4×5000×10-8×106=200; recombination rate ρ=4Nrl=4×5000×10-8×106=200 and no migration.
(DOCX)

Table S12 **We detected the power of the CHI test in different mutation rate θ with certain numbers of loci.**
Other parameters in “steady states” were as follows: sequence length l = 1Mbp; sample size n=400 individuals, in which half of them came from subpopulation 1 and the other half came from subpopulation 2; effective population size N=5000; recombination rate ρ=4Nrl=4×5000×10-8×106=200; divergence time of the two subpopulations is T=10000 years and no migration.
(DOCX)
