# Supplementary material for: A Monte Carlo Permutation Test for Random Mating Using Genome Sequences
Source: PLoS One. 2013 Aug 5;8(8):e71496. doi: 10.1371/journal.pone.0071496 (PMC3734302; doi:10.1371/journal.pone.0071496)
Supplement: Table S1 — We detected type 1 error of the MCP test in different sequence length l corresponding to two different significance levels 0.05 and 0.01. Other parameters in “steady states” were as follows: sample size n=400 individuals; effective population size N=5000; recombination rate ρ = 4Nrl=4×5000×10-8l; mutation rate θ = 4Nμl=4×5000×10-8l. (DOCX) [file pone.0071496.s001.docx]

**Table S1 Type 1 error rate of the MCP test with different sequence length**

| Significance level | *l* = 1kb | *l*= 3kb | *l*= 5kb | *l* =10kb | *l* = 30kb | *l =*50kb |
| --- | --- | --- | --- | --- | --- | --- |
| 0.05 | 0.024 | 0.037 | 0.034 | 0.047 | 0.055 | 0.054 |
| 0.01 | 0.003 | 0.005 | 0.004 | 0.011 | 0.015 | 0.016 |
| Significance level | *l* = 100kb | *l*= 300kb | *l*= 500kb | *l* =1Mb | *l* = 1.5Mb | *l =*2Mb |
| 0.05 | 0.049 | 0.055 | 0.041 | 0.069 | 0.054 | 0.048 |
| 0.01 | 0.006 | 0.013 | 0.004 | 0.016 | 0.008 | 0.015 |
