# Supplementary material for: A Monte Carlo Permutation Test for Random Mating Using Genome Sequences
Source: PLoS One. 2013 Aug 5;8(8):e71496. doi: 10.1371/journal.pone.0071496 (PMC3734302; doi:10.1371/journal.pone.0071496)
Supplement: Table S2 — We detected type 1 error of the MCP test in different sample size n corresponding to two different significance levels 0.05 and 0.01. Other parameters in “steady states” were as follows: sequence length l = 1Mb; effective population size N=5000; recombination rate ρ=4Nrl=4×5000×10-8×106=200; mutation rate θ=4Nμl=4×5000×10-8×106=200. (DOCX) [file pone.0071496.s002.docx]

**Table S2 Type 1 error rate of the MCP test with different sample size**

| Significance level | *n =* 50 | *n =*100 | *n =*200 | *n =* 300 | *n =* 400 |
| --- | --- | --- | --- | --- | --- |
| 0.05 | 0.027 | 0.048 | 0.051 | 0.047 | 0.048 |
| 0.01 | 0.007 | 0.012 | 0.008 | 0.005 | 0.011 |
| Significance level | *n =*500 | *n =*600 | *n =*700 | *n =* 800 | *n =*1000 |
| 0.05 | 0.049 | 0.058 | 0.050 | 0.054 | 0.051 |
| 0.01 | 0.007 | 0.011 | 0.015 | 0.017 | 0.011 |
