# Supplementary material for: A Monte Carlo Permutation Test for Random Mating Using Genome Sequences
Source: PLoS One. 2013 Aug 5;8(8):e71496. doi: 10.1371/journal.pone.0071496 (PMC3734302; doi:10.1371/journal.pone.0071496)
Supplement: Table S3 — We detected type 1 error of the MCP test in different recombination ρ corresponding to two different significance levels 0.05 and 0.01. Other parameters in “steady states” were as follows: sequence length l = 1Mb; effective population size N=5000; mutation rate θ=4Nμl=4×5000×10-8=200; sample size n=400 individuals. (DOCX) [file pone.0071496.s003.docx]

**Table S3 Type 1 error rate of the MCP test with different recombination rate**

| Significance level | ρ = 50 | ρ = 100 | ρ = 200 | ρ = 400 |
| --- | --- | --- | --- | --- |
| 0.05 | 0.037 | 0.048 | 0.051 | 0.039 |
| 0.01 | 0.011 | 0.004 | 0.011 | 0.010 |
| Significance level | ρ = 600 | ρ = 800 | ρ = 1000 |  |
| 0.05 | 0.049 | 0.043 | 0.038 |  |
| 0.01 | 0.010 | 0.007 | 0.005 |  |
