# Supplementary material for: A Monte Carlo Permutation Test for Random Mating Using Genome Sequences
Source: PLoS One. 2013 Aug 5;8(8):e71496. doi: 10.1371/journal.pone.0071496 (PMC3734302; doi:10.1371/journal.pone.0071496)
Supplement: Table S4 — We detected type 1 error of the MCP test in different mutation rate θ corresponding to two significance different levels 0.05 and 0.01. Other parameters in “steady states” were as follows: sequence length l = 1Mb; effective population size N=5000; recombination rate ρ=4Nrl=4×5000×10-8×106=200, sample size n=400 individuals. (DOCX) [file pone.0071496.s004.docx]

**Table S4 Type 1 error of the MCP test with different mutation rate**

| Significance level | θ = 50 | θ= 100 | θ= 200 | θ= 400 |
| --- | --- | --- | --- | --- |
| 0.05 | 0.064 | 0.054 | 0.057 | 0.043 |
| 0.01 | 0.018 | 0.010 | 0.012 | 0.008 |
| Significance level | θ = 600 | θ= 800 | θ= 1000 |  |
| 0.05 | 0.042 | 0.042 | 0.061 |  |
| 0.01 | 0.006 | 0.004 | 0.010 |  |
