# Supplementary material for: A Monte Carlo Permutation Test for Random Mating Using Genome Sequences
Source: PLoS One. 2013 Aug 5;8(8):e71496. doi: 10.1371/journal.pone.0071496 (PMC3734302; doi:10.1371/journal.pone.0071496)
Supplement: Table S5 — We compared the type Ι error rate of the MCP test with the CHI test in different recombination rate ρ corresponding to two different significance levels 0.05 and 0.01. Other parameters in “steady states” were as follows: sequence length l = 1Mb; effective population size N=5000; mutation rate θ=4Nμl=4×5000×10-8×106=200; sample size n=400 individuals. (DOCX) [file pone.0071496.s005.docx]

**Table S5 Comparison of type 1 error between the MCP and CHI tests with different recombination rate**

|  | ρ = 50 | ρ = 100 | ρ = 200 | ρ = 400 | ρ = 1000 |
| --- | --- | --- | --- | --- | --- |
| The MCP^0.05^ | 0.037 | 0.048 | 0.051 | 0.039 | 0.038 |
| The CHI^0.05^ | 0.102 | 0.130 | 0.122 | 0.113 | 0.116 |
| The MCP^0.01^ | 0.011 | 0.004 | 0.011 | 0.010 | 0.005 |
| The CHI^0.01^ | 0.064 | 0.081 | 0.077 | 0.063 | 0.081 |
