# Supplementary material for: A Monte Carlo Permutation Test for Random Mating Using Genome Sequences
Source: PLoS One. 2013 Aug 5;8(8):e71496. doi: 10.1371/journal.pone.0071496 (PMC3734302; doi:10.1371/journal.pone.0071496)
Supplement: Table S6 — We detected the type 1 error of the CHI test in different sequence length l with certain numbers of loci. The longer the sequences, the more loci we could use. Empty cells meant we did not do the experiments because of limited SNPs. Other parameters in “steady states” were as follows: sample size n=400 individual from a random mating population; effective population size N=5000; mutation rate θ=4Nμl=4×5000×10-8×106=200; recombination rate ρ=4Nrl=4×5000×10-8×106=200. (DOCX) [file pone.0071496.s006.docx]

**Table S6 Type 1 error of the CHI with different loci and different sequence length, corresponding to significance level 0.05**

| Sequence | | | | | | Number of loci | | | | | |
| --- | --- | --- | --- | --- | --- | --- | --- | --- | --- | --- | --- |
| length | 1 | 10 | 20 | 30 | 40 | 50 | 60 | 70 | 80 | 90 | 100 |
| 1kb | 0.025 |  |  |  |  |  |  |  |  |  |  |
| 3kb | 0.034 |  |  |  |  |  |  |  |  |  |  |
| 5kb | 0.033 |  |  |  |  |  |  |  |  |  |  |
| 10kb | 0.049 |  |  |  |  |  |  |  |  |  |  |
| 30kb | 0.046 |  |  |  |  |  |  |  |  |  |  |
| 50kb | 0.052 | 0.090 |  |  |  |  |  |  |  |  |  |
| 100kb | 0.052 | 0.106 | 0.120 |  |  |  |  |  |  |  |  |
| 300kb | 0.059 | 0.096 | 0.110 | 0.130 |  |  |  |  |  |  |  |
| 500kb | 0.038 | 0.091 | 0.108 | 0.125 | 0.139 | 0.135 |  |  |  |  |  |
| 1Mb | 0.048 | 0.082 | 0.095 | 0.131 | 0.129 | 0.144 | 0.145 | 0.144 | 0.165 | 0.161 | 0.166 |
| 1.5M | 0.048 | 0.096 | 0.120 | 0.126 | 0.130 | 0.132 | 0.146 | 0.144 | 0.140 | 0.168 | 0.168 |
| 2Mb | 0.052 | 0.102 | 0.102 | 0.107 | 0.123 | 0.131 | 0.140 | 0.134 | 0.145 | 0.138 | 0.136 |
