# Supplementary material for: A Monte Carlo Permutation Test for Random Mating Using Genome Sequences
Source: PLoS One. 2013 Aug 5;8(8):e71496. doi: 10.1371/journal.pone.0071496 (PMC3734302; doi:10.1371/journal.pone.0071496)
Supplement: Table S7 — We detected the type 1 error of the CHI test in different sample size n with certain numbers of loci. Other parameters in “steady states” were as follows: sequence length l = 1Mbp; effective population size N=5000; mutation rate θ=4Nμl=4×5000×10-8×106=200; recombination rate ρ=4Nrl=4×5000×10-8×106=200. (DOCX) [file pone.0071496.s007.docx]

**Table S7 Type 1 error of the CHI test with different loci and different sample size, corresponding to significance level 0.05**

| Sample | | | | | | Number of loci | | | | | |
| --- | --- | --- | --- | --- | --- | --- | --- | --- | --- | --- | --- |
| Size | 1 | 10 | 20 | 30 | 40 | 50 | 60 | 70 | 80 | 90 | 100 |
| 50 | 0.042 | 0.041 | 0.051 | 0.045 | 0.053 | 0.072 | 0.069 | 0.079 | 0.085 | 0.086 | 0.095 |
| 100 | 0.056 | 0.062 | 0.080 | 0.085 | 0.084 | 0.092 | 0.102 | 0.092 | 0.092 | 0.109 | 0.115 |
| 200 | 0.049 | 0.068 | 0.091 | 0.084 | 0.108 | 0.108 | 0.097 | 0.111 | 0.120 | 0.109 | 0.134 |
| 300 | 0.055 | 0.077 | 0.096 | 0.111 | 0.116 | 0.118 | 0.112 | 0.127 | 0.126 | 0.124 | 0.134 |
| 400 | 0.042 | 0.085 | 0.101 | 0.127 | 0.126 | 0.131 | 0.129 | 0.146 | 0.142 | 0.151 | 0.145 |
| 500 | 0.049 | 0.078 | 0.104 | 0.112 | 0.124 | 0.121 | 0.135 | 0.135 | 0.143 | 0.151 | 0.148 |
| 600 | 0.058 | 0.098 | 0.105 | 0.116 | 0.131 | 0.147 | 0.135 | 0.162 | 0.158 | 0.160 | 0.160 |
| 700 | 0.037 | 0.095 | 0.113 | 0.130 | 0.141 | 0.145 | 0.156 | 0.154 | 0.171 | 0.187 | 0.164 |
| 800 | 0.054 | 0.091 | 0.113 | 0.140 | 0.151 | 0.156 | 0.168 | 0.163 | 0.187 | 0.170 | 0.166 |
| 1000 | 0.051 | 0.095 | 0.128 | 0.112 | 0.142 | 0.143 | 0.139 | 0.146 | 0.164 | 0.151 | 0.171 |
