# Supplementary material for: A Monte Carlo Permutation Test for Random Mating Using Genome Sequences
Source: PLoS One. 2013 Aug 5;8(8):e71496. doi: 10.1371/journal.pone.0071496 (PMC3734302; doi:10.1371/journal.pone.0071496)
Supplement: Table S8 — We detected the type 1 error of the CHI test in different mutation rate with certain numbers of loci. Other parameters in “steady states” were as follows: sequence length l = 1Mbp; sample size n=400 individuals from a random mating population; effective population size N=5000; recombination rate ρ=4Nrl=4×5000×10-8×106=200. (DOCX) [file pone.0071496.s008.docx]

**Table S8 Type 1 error of the CHI test with different loci and different mutation rate, corresponding to significance level 0.05**

| Mutation | | | | | | Number of loci | | | | | |
| --- | --- | --- | --- | --- | --- | --- | --- | --- | --- | --- | --- |
| Rate | 1 | 10 | 20 | 30 | 40 | 50 | 60 | 70 | 80 | 90 | 100 |
| 50 | 0.053 | 0.101 | 0.101 | 0.115 | 0.124 | 0.136 | 0.135 | 0.144 | 0.150 | 0.107 | 0.157 |
| 100 | 0.060 | 0.089 | 0.104 | 0.117 | 0.118 | 0.117 | 0.14 | 0.143 | 0.148 | 0.157 | 0.148 |
| 200 | 0.061 | 0.078 | 0.100 | 0.126 | 0.121 | 0.126 | 0.135 | 0.131 | 0.150 | 0.150 | 0.154 |
| 400 | 0.056 | 0.094 | 0.121 | 0.102 | 0.125 | 0.147 | 0.127 | 0.133 | 0.126 | 0.137 | 0.147 |
| 600 | 0.042 | 0.083 | 0.092 | 0.119 | 0.109 | 0.121 | 0.135 | 0.126 | 0.142 | 0.126 | 0.113 |
| 800 | 0.044 | 0.086 | 0.100 | 0.127 | 0.126 | 0.130 | 0.147 | 0.152 | 0.162 | 0.146 | 0.136 |
| 1000 | 0.056 | 0.099 | 0.115 | 0.117 | 0.126 | 0.133 | 0.128 | 0.147 | 0.143 | 0.138 | 0.162 |
