# Supplementary material for: A Monte Carlo Permutation Test for Random Mating Using Genome Sequences
Source: PLoS One. 2013 Aug 5;8(8):e71496. doi: 10.1371/journal.pone.0071496 (PMC3734302; doi:10.1371/journal.pone.0071496)
Supplement: Table S9 — We detected the power of CHI test in different migration rate M=4Nm with certain numbers of loci. (m is the fraction of each subpopulation made up of new migrants each generation.) Other parameters in “steady states” were as follows: sample size n=400 individuals, in which half of them came from subpopulation 1 and the other half came from subpopulation 2; sequence length l = 1Mbp; effective population size N=5000; mutation rate θ=4Nμl=4×5000×10-8×106=200; divergence time T = 10000 years, recombination rate ρ=4Nrl=4×5000×10-8×106=200. (DOCX) [file pone.0071496.s009.docx]

**Table S9 Power of the CHI test with different number of loci and different migration rate, corresponding to significance level 0.05**

| Number of loci | *M* = 0 | *M* = 5 | *M* = 10 | *M* = 20 | *M* = 50 |
| --- | --- | --- | --- | --- | --- |
| 10 | 0.253 | 0.182 | 0.151 | 0.119 | 0.105 |
| 20 | 0.281 | 0.202 | 0.185 | 0.132 | 0.105 |
| 30 | 0.338 | 0.259 | 0.227 | 0.142 | 0.104 |
| 40 | 0.347 | 0.239 | 0.225 | 0.145 | 0.090 |
| 50 | 0.417 | 0.268 | 0.247 | 0.171 | 0.097 |
| 60 | 0.428 | 0.329 | 0.258 | 0.160 | 0.098 |
| 70 | 0.522 | 0.390 | 0.310 | 0.218 | 0.130 |
| 80 | 0.505 | 0.363 | 0.290 | 0.210 | 0.112 |
| 90 | 0.623 | 0.466 | 0.375 | 0.233 | 0.155 |
| 100 | 0.570 | 0.414 | 0.328 | 0.225 | 0.134 |
