# Supplementary material for: A Monte Carlo Permutation Test for Random Mating Using Genome Sequences
Source: PLoS One. 2013 Aug 5;8(8):e71496. doi: 10.1371/journal.pone.0071496 (PMC3734302; doi:10.1371/journal.pone.0071496)
Supplement: Table S10 — We detected the power of the CHI test in different sequence length l with certain numbers of loci. Empty cells meant we did not do the experiments because of limited SNPs. Other parameters in “steady states” were as follows: sample size n=400 individuals, in which half of them came from subpopulation 1 and the other half came from subpopulation 2; effective population size N=5000; mutation rate θ = 4Nμl=4×5000×10-8l; divergence time T = 10000 years, recombination rate ρ = 4Nrl=4×5000×10-8l and no migration. (DOCX) [file pone.0071496.s010.docx]

**Table S10 Power of the CHI test with different loci and different sequence length, corresponding to significance level 0.05**

| Sequence Number of loci | | | | | | | | | | | |
| --- | --- | --- | --- | --- | --- | --- | --- | --- | --- | --- | --- |
| length | 1 | 10 | 20 | 30 | 40 | 50 | 60 | 70 | 80 | 90 | 100 |
| 1kb | 0.094 |  |  |  |  |  |  |  |  |  |  |
| 3kb | 0.082 |  |  |  |  |  |  |  |  |  |  |
| 5kb | 0.092 |  |  |  |  |  |  |  |  |  |  |
| 10kb | 0.072 |  |  |  |  |  |  |  |  |  |  |
| 30kb | 0.074 |  |  |  |  |  |  |  |  |  |  |
| 50kb | 0.077 | 0.125 |  |  |  |  |  |  |  |  |  |
| 100kb | 0.057 | 0.114 | 0.119 |  |  |  |  |  |  |  |  |
| 300kb | 0.054 | 0.096 | 0.144 | 0.148 |  |  |  |  |  |  |  |
| 500kb | 0.086 | 0.093 | 0.124 | 0.145 | 0.159 | 0.211 |  |  |  |  |  |
| 1Mb | 0.081 | 0.118 | 0.150 | 0.150 | 0.183 | 0.183 | 0.195 | 0.216 | 0.195 | 0.200 | 0.215 |
| 1.5Mb | 0.074 | 0.102 | 0.112 | 0.132 | 0.181 | 0.197 | 0.173 | 0.208 | 0.198 | 0.228 | 0.219 |
| 2Mb | 0.074 | 0.099 | 0.170 | 0.161 | 0.171 | 0.179 | 0.202 | 0.225 | 0.216 | 0.246 | 0.232 |
