# Supplementary material for: A Monte Carlo Permutation Test for Random Mating Using Genome Sequences
Source: PLoS One. 2013 Aug 5;8(8):e71496. doi: 10.1371/journal.pone.0071496 (PMC3734302; doi:10.1371/journal.pone.0071496)
Supplement: Table S11 — We detected the power of the CHI test in different sample size n with certain numbers of loci. Other parameters in “steady states” were as follows: sample size n=400 individuals, in which half of them came from subpopulation 1 and the other half came from subpopulation 2; the divergence time of the two subpopulations T=10000 years; effective population size N=5000; mutation rate θ=4Nμl=4×5000×10-8×106=200; recombination rate ρ=4Nrl=4×5000×10-8×106=200 and no migration. (DOCX) [file pone.0071496.s011.docx]

**Table S11 Power of the CHI test with different loci and different sample size, corresponding to significance level 0.05**

| Sample | | | | | | Number of loci | | | | | |
| --- | --- | --- | --- | --- | --- | --- | --- | --- | --- | --- | --- |
| size | 1 | 10 | 20 | 30 | 40 | 50 | 60 | 70 | 80 | 90 | 100 |
| 50 | 0.076 | 0.136 | 0.142 | 0.178 | 0.174 | 0.175 | 0.192 | 0.208 | 0.212 | 0.187 | 0.206 |
| 100 | 0.066 | 0.151 | 0.177 | 0.186 | 0.222 | 0.262 | 0.249 | 0.296 | 0.313 | 0.286 | 0.319 |
| 200 | 0.091 | 0.228 | 0.269 | 0.335 | 0.32 | 0.368 | 0.469 | 0.407 | 0.436 | 0.506 | 0.434 |
| 300 | 0.100 | 0.257 | 0.296 | 0.355 | 0.391 | 0.446 | 0.537 | 0.496 | 0.520 | 0.570 | 0.541 |
| 400 | 0.130 | 0.255 | 0.300 | 0.279 | 0.424 | 0.498 | 0.530 | 0.574 | 0.551 | 0.579 | 0.622 |
| 500 | 0.126 | 0.278 | 0.320 | 0.381 | 0.478 | 0.455 | 0.540 | 0.624 | 0.552 | 0.696 | 0.655 |
| 600 | 0.112 | 0.289 | 0.388 | 0.374 | 0.415 | 0.453 | 0.541 | 0.541 | 0.596 | 0.637 | 0.670 |
| 700 | 0.159 | 0.244 | 0.312 | 0.395 | 0.424 | 0.495 | 0.502 | 0.601 | 0.622 | 0.700 | 0.681 |
| 800 | 0.128 | 0.272 | 0.290 | 0.397 | 0.358 | 0.508 | 0.509 | 0.544 | 0.590 | 0.573 | 0.683 |
| 1000 | 0.142 | 0.258 | 0.378 | 0.494 | 0.490 | 0.565 | 0.608 | 0.63 | 0.684 | 0.728 | 0.753 |
