# Supplementary material for: A Monte Carlo Permutation Test for Random Mating Using Genome Sequences
Source: PLoS One. 2013 Aug 5;8(8):e71496. doi: 10.1371/journal.pone.0071496 (PMC3734302; doi:10.1371/journal.pone.0071496)
Supplement: Table S12 — We detected the power of the CHI test in different mutation rate θ with certain numbers of loci. Other parameters in “steady states” were as follows: sequence length l = 1Mbp; sample size n=400 individuals, in which half of them came from subpopulation 1 and the other half came from subpopulation 2; effective population size N=5000; recombination rate ρ=4Nrl=4×5000×10-8×106=200; divergence time of the two subpopulations is T=10000 years and no migration. (DOCX) [file pone.0071496.s012.docx]

**Table S12 Power of the CHI test with different loci and different mutation rate, corresponding to significance level 0.05**

| Mutation | | | | | | | | | | | | Number of loci | | | | | | | | | | |
| --- | --- | --- | --- | --- | --- | --- | --- | --- | --- | --- | --- | --- | --- | --- | --- | --- | --- | --- | --- | --- | --- | --- |
| rate | | 1 | | 10 | | 30 | | 40 | | 50 | | 60 | | 70 | | 80 | | 90 | | 90 | | 100 |
| 50 | 0.116 | | 0.22 | | 0.324 | | 0.378 | | 0.358 | | 0.493 | | 0.465 | | 0.555 | | 0.532 | | 0.436 | | 0.595 | |
| 100 | 0.125 | | 0.261 | | 0.303 | | 0.345 | | 0.449 | | 0.485 | | 0.506 | | 0.531 | | 0.612 | | 0.603 | | 0.626 | |
| 200 | 0.092 | | 0.27 | | 0.314 | | 0.273 | | 0.401 | | 0.435 | | 0.518 | | 0.507 | | 0.509 | | 0.575 | | 0.579 | |
| 400 | 0.106 | | 0.237 | | 0.284 | | 0.370 | | 0.421 | | 0.465 | | 0.505 | | 0.506 | | 0.623 | | 0.581 | | 0.604 | |
| 600 | 0.139 | | 0.229 | | 0.312 | | 0.394 | | 0.415 | | 0.496 | | 0.507 | | 0.556 | | 0.561 | | 0.640 | | 0.692 | |
| 800 | 0.120 | | 0.211 | | 0.262 | | 0.315 | | 0.402 | | 0.443 | | 0.523 | | 0.477 | | 0.513 | | 0.592 | | 0.653 | |
| 1000 | 0.103 | | 0.208 | | 0.301 | | 0.282 | | 0.429 | | 0.441 | | 0.488 | | 0.506 | | 0.554 | | 0.629 | | 0.601 | |
